# Young and intense: FoxP2 immunoreactivity in Area X varies with age, song stereotypy, and singing in male zebra finches

**DOI:** 10.3389/fncir.2013.00024

**Published:** 2013-02-28

**Authors:** Christopher K. Thompson, Fabian Schwabe, Alexander Schoof, Ezequiel Mendoza, Jutta Gampe, Christelle Rochefort, Constance Scharff

**Affiliations:** ^1^Verhaltensbiologie, Institut für Biologie, Freie UniversitätBerlin, Germany; ^2^Max Planck Institute for Demographic ResearchRostock, Germany; ^3^Neurobiologie des Processus Adaptatifs (UMR 7102), Navigation, Memory, and Aging (ENMVI) Team, Université Pierre et Marie Curie - Centre National de la Recherche ScientifiqueParis, France

**Keywords:** FOXP2, song system, Bayesian statistics, striatum, basal ganglia, language, DVD, apraxia

## Abstract

FOXP2 is a transcription factor functionally relevant for learned vocalizations in humans and songbirds. In songbirds, FoxP2 mRNA expression in the medium spiny neurons of the basal ganglia song nucleus Area X is developmentally regulated and varies with singing conditions in different social contexts. How individual neurons in Area X change FoxP2 expression across development and in social contexts is not known, however. Here we address this critical gap in our understanding of FoxP2 as a link between neuronal networks and behavior. We used a statistically unbiased analysis of FoxP2-immunoreactivity (FoxP2-IR) on a neuron-by-neuron basis and found a bimodal distribution of FoxP2-IR neurons in Area X: weakly-stained and intensely-stained. The density of intensely-stained FoxP2-IR neurons was 10 times higher in juveniles than in adults, exponentially decreased with age, and was negatively correlated with adult song stability. Three-week old neurons labeled with BrdU were more than five times as likely to be intensely-stained than weakly-stained. The density of FoxP2-IR putative migratory neurons with fusiform-shaped nuclei substantially decreased as birds aged. The density of intensely-stained FoxP2-IR neurons was not affected by singing whereas the density of weakly-stained FoxP2-IR neurons was. Together, these data indicate that young Area X medium spiny neurons express FoxP2 at high levels and decrease expression as they become integrated into existing neural circuits. Once integrated, levels of FoxP2 expression correlate with singing behavior. Together, these findings raise the possibility that FoxP2 levels may orchestrate song learning and song stereotypy in adults by a common mechanism.

## Introduction

FOXP2^1^ (Kaestner et al., 2000) is a transcription factor that is essential for the accurate acquisition of learned vocalizations in humans (Lai et al., 2001). FOXP2 expression during human brain development coincides with adult sites of pathology, among them cortico-cerebellar and cortico-striatal circuits (Lai et al., 2003; Liegeois et al., 2003). Songbirds have helped address the role of FoxP2 in the development and function of the neural circuits involved in learned vocalizations. Like speech, song of zebra finches and other songbirds is imitatively learned, and the acquisition, as well as maintenance, of these learned vocalizations depends upon auditory information (Doupe and Kuhl, 1999). Song learning is mediated by a cortico-striato-thalamo-cortical circuit known as the anterior forebrain pathway (AFP) (Nottebohm et al., 1976; Scharff and Nottebohm, 1991), which has anatomical and functional similar features to the mammalian equivalent (Reiner, 2002; Fisher and Scharff, 2009). The basal ganglia component of the AFP is called Area X, which is composed of striatal medium spiny neurons, interneurons, and a more pallidal-like neuron type (Person et al., 2008). Area X receives afferents from pallial song nucleus HVC (Okuhata and Saito, 1987), and via a thalamic relay projects back to a pallial nucleus (lMAN) before connecting to the song production motor pathway (Bottjer et al., 1989; Vates and Nottebohm, 1995). Area X expresses FoxP2 at high levels in a subset of the striatal medium spiny neurons (Haesler et al., 2004; Teramitsu et al., 2004). Other Area X interneurons, such as cholinergic neurons and neurons positive for nitric oxide synthase and parvalbumin, do not express FoxP2 (Haesler et al., 2004; Rochefort et al., 2007). Likewise, the pallidal-like projection neurons that synapse onto thalamic neurons do not express FoxP2 (Rochefort et al., 2007). FoxP2 is expressed in the proliferative ventricular zone from which Area X recruits new neurons, and more new FoxP2-IR neurons are recruited to Area X in juveniles than in adults (Rochefort et al., 2007). *FoxP2* mRNA expression in Area X is higher than in the surrounding striatum at 35 and 50 days after hatching (i.e., post-hatch day, PHD), the age when song is learned, and decreases once birds reach sexual maturity and song learning has ended (Haesler et al., 2004), suggesting that FoxP2 plays a role in mediating song learning and vocal plasticity. Indeed, experimental downregulation of FoxP2 shows that adequate FoxP2 levels are necessary for correct acquisition of song in young zebra finches (Haesler et al., 2007). Combined findings from songbirds and mice suggest that FoxP2 may influence motor learning by regulating dendritic spine density (Schulz et al., 2010), synaptic plasticity (Groszer et al., 2008; Enard et al., 2009; French et al., 2011) and neurite growth and/or maintenance (Vernes et al., 2011). In songbirds, FoxP2 appears to contribute to neural circuit function in adults as well since song rate and social context alter expression of FoxP2 mRNA and protein in Area X at a minute to hour time scale. Specifically, after males sing to themselves (called undirected song) during a 2 h period, there is less FoxP2 mRNA expressed in Area X than when birds engage in courtship song (called directed song) for the same amount of time (Teramitsu and White, 2006). In contrast, immediate early genes, including the transcription factors egr-1 (also called zenk) and c-fos, are upregulated in Area X as a result of undirected song but not after directed singing (Jarvis et al., 1998; Tokarev et al., 2011).

Changes in FoxP2 expression during the maturation and aging process of adult-born neurons have not been explored. This is important missing information to link what we know from developmentally and behaviorally regulated global expression changes across all Area X neurons on the one hand, and from genetic manipulations to reduce FoxP2 in a subset of Area X tissue on the other hand to the role of FoxP2 levels in individual Area X neurons.

Given that in mice Foxp2 levels are linked to striatal synaptic plasticity, and that Foxp2, in synergy with Foxp4, is required for the normal progression of neural precursors into mature neurons (Rousso et al., 2012; Tsui et al., 2013), we hypothesized that different levels of FoxP2 expression in individual Area X neurons may be associated with different stages of their neural maturation and consequently be associated with different functions during song learning and adult song plasticity. Here we address the relationship between changing levels of FoxP2 protein expression in individual striatal neurons at various maturational stages and at different ages of the bird's life and relate these findings to song development and song stability.

## Materials and methods

All procedures were approved by the institutional animal care and use committee.

### Behavioral conditions and song recordings

We used male zebra finches from the Department of Animal Behavior at the Freie Universität in Berlin. The colony was kept under a constant 12:12 light/dark cycle, and food and water were provided *ad libitum.* To evaluate FoxP2-IR in juveniles, males at PHD 35 (*n* = 4) or PHD 50 (*n* = 3) were taken directly out of their home cages between 2 and 6 h after lights turned on in the morning and overdosed with inhalant anesthesia (see below). Juvenile singing behavior was not monitored beforehand. To evaluate FoxP2-IR in adults, we recorded song of individual adult males (aged between PHD 133 and 3000) in soundproof chambers. One male was of unknown age when acquired 8 years before being used in this study and thus had an estimated age of 3000 days. All other males were born in the FU colony and of known age. Song was continuously recorded via an omni-directional microphone attached to a computer running Sound Analysis Pro (SAP) 1.04 (Tchernichovski et al., 2000). Lights in the recording boxes went on at 8:00 AM. and were set to 14:10 L:D in order to replicate the photoperiod regime used in previous experiments on rapid modulation of FoxP2 expression as a result of singing in zebra finches (Teramitsu and White, 2006; Miller et al., 2008; Teramitsu et al., 2010).

The animals were divided into four groups, following methods used previously (Miller et al., 2008): one group of males was euthanized in the morning immediately after lights went on and therefore did not sing (non-singers, NS 0 h, *N* = 8). In the 2 h following lights-on, three groups of males were treated in the following manner: males in one group did not sing or were kept from singing by the presence of an investigator (non-singers, NS 2 h, *N* = 5), in a second group males sang toward conspecific females (directed singers, Dir, *N* = 6), and in the last group males sang by themselves (undirected singers, Undir, *N* = 7). Each male in the Dir group was visually and acoustically exposed to a different female every 4–8 min to keep singing rates high. Females were held in separate transparent chambers attached to the front of the male cage. We considered males as singers (Dir and Undir) when they sang at least 90 motifs within 2 h after lights on. Birds were considered to be non-singers if they sang less than 10 motifs (Miller et al., 2008), but none of the birds in the NS 2 h groups in this study sang any bouts.

### BrdU injections

Dividing cells were labeled by intramuscular injection with the DNA synthesis marker, 5-bromo-20-deoxyuridine (BrdU, 50 mg/kg of body weight, dissolved in 0.4 N NaOH with 0.9% NaCl). Male juvenile zebra finches received five BrdU injections (one every 2 h) at 29 and 79 PHD and were euthanized at 50 and 100 PHD, respectively. Results from these animals were used in a previous study (Rochefort et al., 2007). To assess the potential relationship of FoxP2 to neuronal migration, we treated two adults with four BrdU injections (one every 2 h) and overdosed them 10 days later.

### Perfusion, preparation of tissue, cresyl violet staining

All birds were overdosed by isofluorane inhalation, followed by transcardial perfusion with heparinized saline and subsequent 4% phosphate-buffered paraformaldehyde (PFA, pH 7.4). Brains were dissected from the skull and transferred it into PFA at 4°C overnight. To cryoprotect the tissue, we sank the brains in 15% sucrose at 4°C then transferred them to 20% sucrose, usually overnight, until sectioning.

Brains were cut in the coronal plane at 40 μm using a freezing microtome (Leica SM 2000 R), every third section was mounted onto chromalum-coated slides and air dried overnight. The tissue was delipidized by passing the slides through two baths of xylene, then through a descending series of ethanol to rehydrate the tissue before transferring them into distilled water. We stained the sections in 0.5% cresyl violet solution, dehydrated them through an ascending series of ethanol (including one 95% ethanol wash with 0.3% acetic acid), cleared the sections in xylene and coverslipped them using DPX (Agar-Scientific Ltd), and air dried the slides overnight prior to analysis. We transferred the remaining sections of interest into trays filled with cryoprotectant (30% ethylene glycol and 30% sucrose in 0.1 M PB) and stored them at −20°C until needed for immunohistochemistry (IHC) (see below).

### FoxP2 IHC

To visualize expression of FoxP2 protein, we performed IHC. Briefly, floating brain sections were washed in 0.1 M phosphate buffered saline (PBS) containing 0.1% Triton X-100 (PBS-TX, pH 7.4) for 15 min six times and then blocked for 1 h in blocking buffer containing 0.4% TX-100, 3% bovine serum albumin, 5% normal horse serum, 0.1% Na azide in PBS. Afterwards, we incubated the sections overnight with a primary antibody against FoxP2 (1:2000, AbCam, ab1307, made in goat) in blocking buffer in 4°C (see “Materials and Methods” for characterizing this antibody below). We then washed the sections in PBS-TX followed by an endogenous peroxidase inhibition using 3% H_2_O_2_, in 10% MeOH and PBS, then, washed the tissue in PBS-TX before incubating them for 1 h with biotinylated secondary antibody in 0.1 M PBS (1:200). We washed the sections in PBS-TX, placed them into an avidin-biotin-complex (ABC) solution (Vectastain ABC Kit, Vector Laboratories) for 40 min, washed in PBS-TX, then PBS, followed by 125 mM Na acetate. Antibody was visualized by labeling by a nickel-enhanced 3,3'-diaminobenzidine (DAB) reaction consisting of 3.3 ml 8% nickel ammonium sulfate, 6.4 ml 196 mM sodium acetate, 100 μl 1 M imidazole, 100 μl 4.5% DAB and 100 μl 3% H_2_O_2_ (Wang and Rubel, 2008). We washed sections in 125 mM Na acetate followed by distilled water, mounted the tissue on chromalum-subbed slides, and let them air dry overnight. Slides were coverslipped with DPX. To minimize the influence of procedural factors on staining intensity across treatment groups, IHC for FoxP2 was done in batches on brain sections from 4 to 6 birds at a time. At least one animal from each behavioral group was included in each batch.

### Characterization of FoxP2 antibody

**Figure 1:**
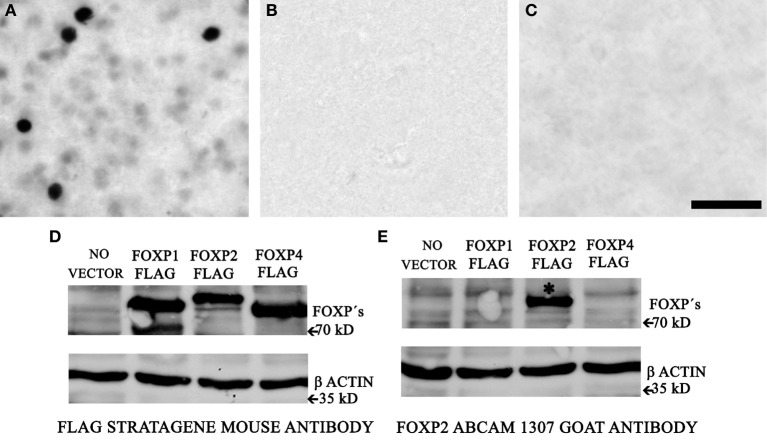
**AbCam 1307 anti-FoxP2 antibody specifically detects FoxP2 in Area X. (A)** Immunohistochemistry using the AbCam 1307 anti-FoxP2 (made in goat) antibody and a biotinylated anti-goat secondary antibody revealed with a nickel-enhanced DAB reaction in a brain section containing Area X from a non-singing adult bird. **(B)** Following the identical procedure as above but omitting the primary AbCam 1307 antibody eliminates staining. **(C)** Pre-incubating the AbCam 1307 antibody with FoxP2 protein prior to IHC also eliminates staining in Area X. Scale bar = 50 μm. **(D)** Western blot of protein extracts from HeLa cells without vector or overexpressing FLAG-tagged FoxP1, or FLAG-tagged FoxP2, or FLAG-tagged FoxP4 probed with an anti-FLAG antibody detects the FLAG epitope of the tagged FoxP proteins. **(E)** Western blot of the same cell extracts as in **(D)** using the AbCam 1307 anti-FoxP2 antibody specifically detects FoxP2 (asterisk) but not FoxP1 or FoxP4.

Our FoxP2 IHC protocol using the AbCam ab1307 FoxP2 antibody labeled nuclei, some of which were intensely-stained and many of which were weakly-stained (Figure 1A). To verify the specificity, we undertook three experiments. First, we prepared negative control sections by leaving out the primary antibody; these control sections showed no staining (Figure 1B). Second, we incubated the primary antibody with FoxP2 protein extracted from cells overexpressing FoxP2 for 1 h at 37°C and then proceeded with the IHC protocol described above. Sections from these experiments showed no staining (Figure 1C). Last, we performed Western blot for FoxP2 (methods described in next section).

### Generation of tagged overexpression plasmids and cotransfection assays to determine specificity of antibodies

To overexpress FoxP1 (accession number AY549152) and FoxP2 (accession number AY549148, AY549149, AY549150, and AY549151) we used V5-tagged expression plasmids described previously (Haesler et al., 2007), designed primers that placed the FLAG-tag before the stop codon, and inserted the products into pcDNA3.1 (+) vectors (Invitrogen). For FoxP4 we designed primers to amplify the entire coding region of zebra finch FoxP4 adding the FLAG-tag before the stop codon followed by an EcoRI restriction site in the rev primer and in the forward primer BamHI restriction site followed by Kozak sequence and the start of FoxP4 (5'-GGATCCGCCACCATGATGGTTGAATCCGCCTCG-3' forward and 5'-GCGGAATTCCTACTTATCGTCGTCATCCTTGTAATCGGACAAGTCTTCCACCGGCAGCTC-3' reverse). The resulting PCR product was examined on an agarose gel, cleared of nucleotides with the Qiaquick PCR purification kit (Qiagen, Chatsworth, CA), cut with the restriction enzymes, and cloned into the pcDNA3.1 (+) vector (Invitrogen). Inserts from four independent FoxP4 clones were then sequenced on both strands. We have deposited the zebra finch FoxP4 sequences to GenBank (accession number NCBI JN160732).

### Western blotting

HeLa cells transiently transfected with lipofectamine (Invitrogen, 11668-019) were lysed with MPER medium for 15 min on ice, then sonicated for 30 s. Extracts were centrifuged for 10 min at 1500 g and the supernatant was dissolved in Laemmli buffer and denatured for 5 min at 95°C. 15 μl of each cell extract were separated by SDSPAGE (10%), transferred to a polyvinylidene fluoride membrane (Roche, Indianapolis, IN), and blocked with 1% RotiBlock (Roth A151) in water overnight at 4°C. The membranes were then incubated overnight at 4°C with the goat FoxP2 (Abcam, ab1307) or Flag M2 (Stratagene 200472) and actin (Sigma, A2066) antibodies at a dilution of 1:2000. After three washes in PBS/0.1%Tween 20 membranes were incubated with an HRP-conjugated antibody raised against the appropriate animal (1:2000 dilution; Amersham Biosciences) for another 30–120 min at RT. Binding was detected on x-ray films using an ECL detection system for HRP (Perkin-Elmer, Boston, MA). The antibody against FlagM2 detected proteins of the expected size in all three FoxP-overexpressing cell lines (Figure 1D). The AbCam anti-FoxP2 antibody specifically labeled FoxP2 but not FoxP1 or FoxP4 in protein extracted from these same cells (Figure 1E).

### FoxP2, BrdU, and vimentin IHC

BrdU-IR cells and vimentin-IR processes were identified using a fluorescent IHC protocol. Double and triple labeling was carried out sequentially, to minimize cross-reactivity. Briefly, we used free-floating 30-μm sections that were permeabilized with 0.2% TritonX-100 and DNA was denatured with HCl (2 N, 30 min at 37°C). We incubated the tissue first with a rat anti-BrdU primary antibody (1:200, ImmunologicalsDirect) overnight. After BrdU detection with Alexa 568-labeled anti-rat secondary antibody (1:500, Molecular Probes), we fixed the brain sections in PFA for 10 min and then incubated them overnight with a rabbit anti-FoxP2 antibody (1:1000, kindly provided by E.E. Morrisey, University of Pennsylvania, Philadelphia) alone or with a mouse anti-vimentin antibody (1:1000, 40E-C, Hybridoma Bank). The antibody against vimentin was originally generated for use in songbirds (Alvarez-Buylla et al., 1987). In sections treated only with antibodies against BrdU and FoxP2, FoxP2-IR was revealed with an Alexa 488-labeled anti-rabbit secondary antibody (1:200; Molecular Probes). For triple label, we used a biotinylated anti-rabbit antibody coupled with an Alexa 350-labeled streptavidin complex (Molecular Probes) to label FoxP2 and an Alexa 488-labeled anti-mouse secondary antibody (1:200; Molecular Probes) to label vimentin. All slides were coverslipped with Mowiol (Calbiochem).

### Analysis of FoxP2-immunoreactivity

Close inspection of the FoxP2-stained adult brain sections revealed that there were two types of neurons that could be distinguished by intensity of IR (Figure 1A). A few neurons with relatively large nuclei were intensely-stained for FoxP2, and many neurons with relatively small nuclei were weakly-stained for FoxP2. We called these types “intensely-stained FoxP2-IR neurons” and “weakly-stained FoxP2-IR neurons.” In order to assess whether these two types constituted separate classes, we quantified the intensity of IR. We examined the sections under a Zeiss Axiovert S100 inverted microscope with 400 fold magnification and took a series of images across the z-axis in order to ensure that measured nuclei were in focus. FoxP2 intensity did not vary as a function of z-axis position. We examined one field per section in six to eight sections per animal across the rostral-caudal extent of Area X and measured all observable FoxP-IR round-shaped nuclei that fell within a 62 μm by of 62 μm field of view. In the non-singing males this resulted in ~20–30 measurements of FoxP2-IR nuclei per field. The average gray value was determined within a 12.9 μm^2^ circular selection window placed over the center of each nucleus. This selection window was small enough to fit within the perimeter of both weakly and intensely-stained nuclei. We did not measure FoxP2-IR nuclei that were substantially smaller than 12.9 μm^2^. Any cell that did not have a spherical appearance, such as fusiform-shaped nuclei, was not measured in this initial analysis (see below for details on fusiform-shaped nuclei analysis). To account for background staining differences, we measured the average gray value of an area of nidopallium dorsal and lateral to the striatum, which had substantially fewer FoxP2-IR nuclei. We carefully selected an area free of FoxP2-IR neurons and repeated this process in 3–4 sections. Any measurements of nuclei that were stained at background levels or only 5% above background were eliminated from analysis. This resulted in removing on average ~4% of all measured nuclei. However, including even the most lightly-stained neurons by analyzing the same data including all measurements stained higher than background (without the added 5% cutoff) returned the same statistical differences across groups.

**Figure 2:**
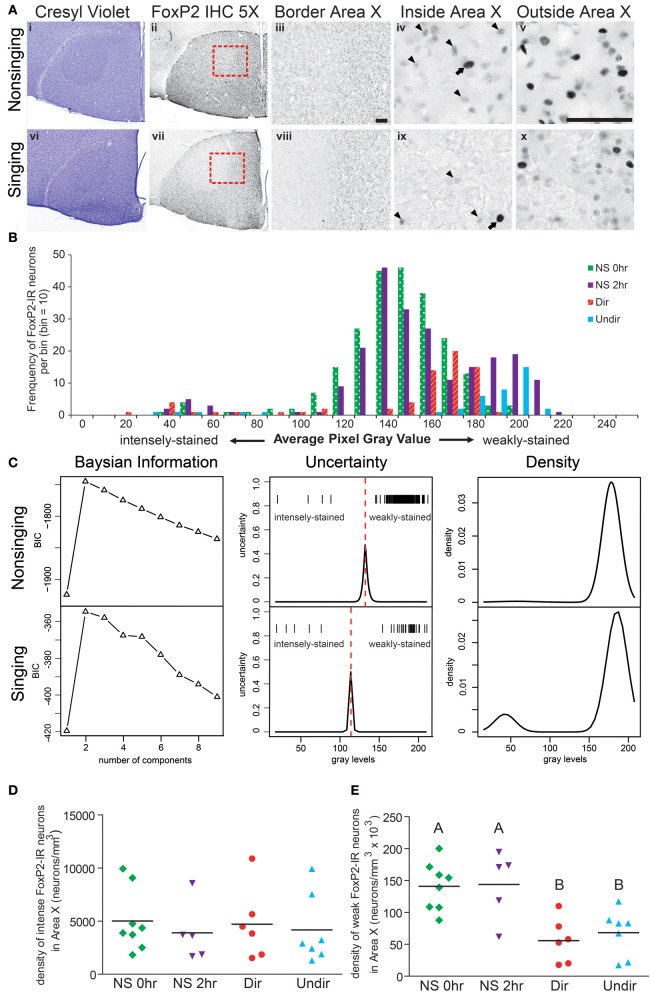
**Singing behavior decreases the density of weakly-stained FoxP2-IR neurons in Area X in adult male zebra finches. (A)** Area X stands out as pear-shaped, darker stained structure in photomicrographs of cresyl violet stained coronal brain sections of a bird that did not sing for 2 h following lights on [Non-singing, (i)] and a bird that sang toward a female [Singing, (vi)]. (ii and vii) Adjacent sections to the cresyl violet stained ones in panels (i and vi), immunolabled for FoxP2. (iii and viii) Higher magnification of the border region of Area X and striatum [red boxes in panels (ii) and (vii)] shows staining inside Area X darker in the non-singing condition (iii) than in the singing condition (viii). Still higher magnification reveals intensely-stained (arrows) and weakly-stained (arrowheads) FoxP2-IR nuclei inside (iv and ix) and outside (v and x) Area X. Scale bar = 50 μm. **(B)** Histograms for Area X FoxP2-IR gray values of four birds, one from each behavioral treatment prior to anesthesia overdose. Note that the majority of measured FoxP2-IR neurons were weakly-stained and that the left tail consists of the less frequent intensely-stained FoxP2-IR neurons. **(C)** Bayesian information analysis indicated that the distributions of FoxP2 immunoreactivity from a non-singing and singing bird were most likely to consist of two components, as assessed by Bayesian information criterion (BIC). The uncertainty in distinguishing between these two cell populations (middle panels) fell under a narrow window, and the peak of the uncertainty served as a cutoff (dotted red line) between intensely and weakly-stained distributions (gray level of each cell = one tick mark). The estimated mixture distribution of staining intensities for these two birds was bimodal (right panels), but note that the vast majority of FoxP2-IR cells in the non-singing animals was weakly-stained, making the relative contribution of intensely-stained cells to the mixture model almost invisible (top right panel). **(D)** The density of intensely-stained FoxP2-IR neurons was not affected by singing behavior (ANOVA, *p* = 0.916). **(E)** Birds that did not sing (NS 0 h and NS 2 h) had significantly higher densities of weakly-stained FoxP2-IR neurons than birds that sang directed (Dir) or undirected (Undir) song (*p* = 0.0015, *post-hoc* differences indicated by letters above columns).

**Figure 3:**
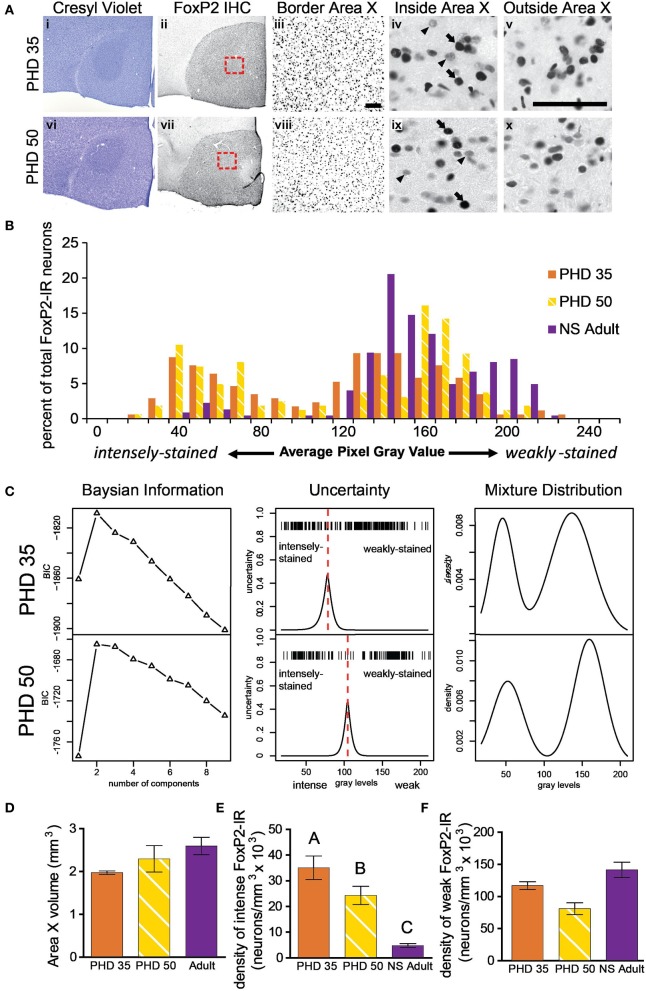
**The proportion of intensely to weakly-stained FoxP2-IR neurons varies between adult and juvenile males. (A)** Photomicrographs of coronal brain sections stained for cresyl violet to visualize Area X in juveniles euthanized at 35 (i) and 50 (vi) post hatch days (PHD). (ii and vii) Adjacent sections to the cresyl violet stained ones in panels (i and vi), immunolabled for FoxP2. (iii and viii) A magnified view of the red boxes in panels (ii and vii), which is placed on the border of Area X and striatum. A magnified view of intensely (arrows) and weakly-stained (arrowheads) FoxP2-IR neurons inside (iv and ix) and outside (v and x) Area X. Scale bar = 50 μm. **(B)** Histograms for FoxP2-IR gray values for three birds: a PHD 35 male, a PHD 50 male, and a NS 2 h adult. Note that the two juveniles show a bimodal distribution of gray values, whereas in the non-singing adult the majority of cells was weakly-stained and a much smaller proportion was intensely-stained. **(C)** Bayesian information analysis finds that two components most likely make up the distributions of FoxP2 immunoreactivity in two representative example juvenile birds, as assessed by Bayesian information criterion (BIC). The uncertainty in distinguishing between these two cell populations falls under a narrow window in both animals (red dotted line), and the peak of the uncertainty serves as a cutoff between intensely and weakly-stained distributions. The estimated mixture distribution for juvenile birds was clearly bimodal. **(D)** The volume of Area X did not significantly vary across these ages (ANOVA, *p* = 0.1191). **(E)** The density of intensely-stained FoxP2-IR neurons was ~10X higher in juveniles than in adults (*p* < 0.0001, *post-hoc* differences indicated by letters above columns). PHD 35 males had significantly greater FoxP2-IR density than PHD 50 males. **(F)** The density of weakly-stained FoxP2-IR neurons did not significantly differ across ages, though there was a trend (*p* = 0.0578).

For each animal, we made histograms of the gray levels measured for FoxP2-IR nuclei (e.g., Figures 2B, 3B). Inspection of the adult data showed that that the majority of nuclei were weakly-stained, distributed in an overall unimodal fashion, and only a small population of intensely-stained nuclei made up the left tail of the distribution (Figure 2B). In contrast, the distribution of gray values in juvenile animals was bimodal (Figure 3B). In order to objectively classify FoxP2-IR neurons as weakly-stained or intensely-stained, we used a mixture-model-based cluster analysis (Fraley and Raftery, 2002). The distribution of gray-values for each individual bird was assumed to stem from a mixture of normally distributed components. The optimal number of components (K) was determined by the Bayesian Information Criterion (BIC), and the parameters of the optimal model were estimated by an Expectation Maximization (EM)-algorithm. We classified each neuron into the category for which its membership probability was highest, using the peak of uncertainty as a cutoff between weakly-stained and intensely-stained neurons for each individual animal (red dotted line, Figures 2C, 3C, middle panel). Additionally, we determined the classification uncertainty for each gray value, which is the complement to the maximum membership probability. All analyses were performed using the R-package MCLUST (Fraley and Raftery, 2006). To determine the density of weakly-stained FoxP2-IR neurons, we counted only those neurons that fell into the weakly-stained neuron category and had gray values higher than the 5% above background cut-off described above.

The above procedure yielded counts of sufficient numbers of weakly-stained FoxP2-IR neurons per animal to calculate the density of weakly-stained FoxP2-IR neurons. Yet there were often only a few intensely-stained FoxP-IR nuclei observed per adult. To obtain more reliable measures of intensely-stained FoxP2-IR neuron density we analyzed FoxP2-IR brain sections using a rectangular field of view of 128 × 162 μm and counted only those neurons that were intensely-stained for FoxP2. We used the same procedure to count just fusiform-shaped FoxP2-IR nuclei; cells with a fusiform appearance are putative migratory, young neurons (Alvarez-Buylla and Nottebohm, 1988). We did not systematically measure the intensity of fusiform shaped nuclei but observed that they were usually more intensely-stained for FoxP2 than weakly-stained FoxP2 IR non-fusiform nuclei. We chose random fields within the borders of Area X using a macro in Image J with the same magnification settings described above. We repeated this process in 2–3 fields in five sections from each brain. All analyses were done blind with respect to treatment group.

### Analysis of BrdU and FoxP2-immunoreactivity

BrdU-IR and FoxP2-IR sections were analyzed with a 40× oil objective using a Zeiss confocal microscope (LSM510) equipped with lasers Ar 488 and HeNe1 543, with LSM-510 software package for image acquisition and data analysis. Pinholes were optimally set at 94 μm for channel 1 and 106 μm for channel 2. Lateral and z-axis resolutions were 0.45 and 1 μm, respectively. We phenotyped BrdU-IR cells within Area X by analyzing in three dimensions reconstructed BrdU-IR nuclei in the x–z and y–z orthogonal projections for the presence or absence of FoxP2-IR. We generated z-projections of confocal stacks from which there was little overlap of BrdU and FoxP2-IR and measured the brightness level in the channel under which only FoxP2-IR was visible. Using a procedure identical to the one described above, we categorized FoxP2-IR nuclei as either intensely-stained or weakly-stained.

### Song analysis

#### Song structure definitions

A “motif” is defined as repeatedly occurring sequences of song elements called “syllables,” which in turn are composed of “notes,” the simplest continuous sound elements that the birds produce during singing. Each bird can have a small variety of motif variants, a “long motif” occurring most of the time and occasional “short” motifs, omitting some notes. A “strophe” is one or (usually) more motifs strung together with pauses no longer than a couple hundred milliseconds. Often a strophe ends with a short motif. A song “bout” is a combination of motifs and/or strophes with silent intervals no longer than 2 s (Brainard and Doupe, 2001).

#### Measurements

To assess the amount of Dir and Undir song sung in the 2 h before being euthanized, we quantified the number of motifs sung using the program Syrinx (Burt, University of Washington) from songs recorded using SAP 1.04 (Tchernichovski et al., 2000).

We measured bioacoustic features of syllables using SAP. We quantified the duration, pitch, frequency modulation, amplitude modulation, entropy, pitch goodness, and average frequency of each syllable in the first motif from the first 20 bouts sung from 20 birds (six birds did not sing enough undirected songs for analysis) recorded on one of the 2 days prior to sacrifice. We also calculated average inter-syllable duration for 20 motifs and the coefficient of variation as follows; the durations of all syllables in a motif were summed up, which was substracted from the duration of the entire motif, then divided by the number of inter-syllable intervals.

Stereotypy of production was measured within a bout and across bouts using the similarity measure in SAP. Within a bout we measured the similarity between the first motif and the third motif in 15 different bouts, similar to Pytte et al. (2007) who compared the first and second motif to the fifth and sixth motif; we compared the first and third motifs because not enough birds sang six or more motifs in a bout. We also measured the duration of the first and third motif, given that motif duration tends to lengthen during a bout (Chi and Margoliash, 2001; Glaze and Troyer, 2006) and that this increase in motif duration is known to be greater in younger adult males than in older males (Pytte et al., 2007).

To compare stereotypy of production across bouts, we compared the SAP similarity values of the first motif of one bout with the first motifs of the preceding and following bout. The similarity from these two comparisons was then averaged, and the process was repeated for 15 different bouts.

In order to analyze the sequence of syllables within motifs, we measured linearity and consistency of 20 strophes using the methods of Scharff and Nottebohm (1991). We calculated linearity by counting the number of different syllables in the motif and dividing it by the number of transition types in the motif. Consistency was calculated by summing all of the typical transitions in a song and dividing it by the sum of all transitions. An overall metric for this analysis, termed sequence stereotypy, was calculating by averaging linearity and consistency.

### Statistical analyses

ANOVA was used to determine whether there were significant differences in the density of weakly and intensely-stained FoxP2-IR neurons between juveniles and adults and across behavioral groups within adults. *Post-hoc* comparisons were calculated with Tukey's Multiple Comparison to identify significant differences between groups. The density of FoxP2-IR neurons in Area X as a function of age was analyzed by one-phase decay non-linear regression analysis. We compared some measures of song characteristics and density of FoxP2-IR neurons to age using an appropriate non-linear regression (e.g., Figures 5A,C). For all other correlations, we used linear regression analysis. For analysis of BrdU and FoxP2-IR colocalization, we used Fisher's Exact Test on a bird-by-bird basis, using presence or absence of BrdU-IR as one category and weakly or intensely-stained FoxP2-IR as another category. Statistical methods for distinguishing weakly-stained from intensely-stained FoxP2-IR neurons are described above.

## Results

### IHC for FoxP2 revealed two categories of FoxP2-IR neurons in adult area X: intensely-stained and weakly-stained

To determine whether singing behavior and/or social context affected the distribution of FoxP2-IR neurons in Area X, we measured the density of intensely and weakly-stained FoxP2-IR neurons in adult male zebra finches that experienced four different behavioral conditions before being overdosed with inhalant anesthesia. We chose these conditions to allow comparison with a previous study on FoxP2 protein expression in Area X (Miller et al., 2008). When lights turned on in the morning, (1) birds did not sing and were immediately overdosed (called NS 0 h), (2) birds did not sing for 2 h and were overdosed at the end of the 2 h (NS 2 h), (3) birds sang to females during the 2 h (Dir), and (4) birds sang while alone during the 2 h (Undir). In all groups, there were a few Area X neurons with relatively large nuclei that were intensely immunoreactive for FoxP2 (arrows in Figure 2A) whereas the majority of FoxP2-IR neurons in adult Area X had small nuclei that were weakly-stained in a punctate pattern (arrowheads in Figure 2A). To gauge whether these intensely- and weakly-stained neurons belonged to the same population of neurons, we prepared histograms of staining intensity measures for each animal. These revealed an apparently unimodal distributions of weakly-stained FoxP2-IR neurons and a left tail consisting of the intensely-stained FoxP2-IR neurons. Representative examples of one individual from each of the four behavioral groups are shown in Figure 2B.

### Bayesian analysis provides an objective method for discriminating weakly-stained FoxP2-IR neurons from intensely-stained FoxP2-IR neurons

Bayesian information analysis (see “Materials and Methods”) confirmed the visual impression that the distribution of gray values for FoxP2-IR nuclei consisted of two distinct components in most individuals (two examples illustrated in Figure 1C, first column). In the few individuals where staining intensities were best explained by three components, two of the three components were non-systematic subdivisions of the weakly-stained FoxP2-IR neurons.

The uncertainty differentiating the intensely-stained population from the weakly-stained population (Figure 2C, second column) fell under a relatively small window, and the peak of uncertainty served as boundary between the populations. Bayesian information analysis was performed for each adult, returning unbiased numbers of intensely-and weakly-stained cells. In all adults examined, intensely-stained FoxP2-IR neurons were rare. In non-singing (NS) adults, the number of intensely-stained FoxP2-IR neurons (i.e., the left tail of the distribution) was very small relative to the much more numerous weakly-stained neurons, making the relative distribution of intensely-stained FoxP2-IR difficult to see (Figure 2C, third column, top row).

### Weakly-stained FoxP2-IR neurons in area X are less numerous in adult male zebra finches after singing

The density of intensely-stained FoxP2-IR neurons in Area X did not vary significantly across behavioral regimes (ANOVA, *p* = 0.9158, *F* = 0.1696, Figure 2D). In contrast, the density of weakly-stained FoxP2-IR neurons in Area X significantly varied across behavioral regimes (ANOVA, *p* = 0.0015, *F* = 7.340 Figure 2E). *Post-hoc* tests showed that the density of weakly-stained FoxP2-IR neurons was significantly greater in the NS 0 h and NS 2 h groups than in the Dir and Undir groups (Figure 2E).

### Density of FoxP2-IR neurons in area X varies as male zebra finches sexually mature

To determine if the density of FoxP2-IR neurons in Area X changes as male zebra finches sexually mature, we measured the density of FoxP2-IR in juveniles (PHD 35 and 50). Intensely-stained FoxP2-IR neurons were more numerous in juveniles (Figure 3A) than in adults (Figure 2A). Histograms and Bayesian information analysis for the degree of immunostaining revealed that FoxP2-IR neurons in PHD 35 and PHD 50 animals could be clearly separated into two categories (Figures 3B,C). Area X volume did not differ significantly across ages (ANOVA, *p* = 0.1191, *F* = 2.720, Figure 3D). We compared the juvenile groups to the NS adult groups because singing reduces the number of weakly-stained FoxP2-IR neurons. The density of intensely-stained FoxP2-IR neurons in Area X varied significantly across ages (ANOVA, *p* < 0.0001, *F* = 214.0, Figure 3E). The density of FoxP2-IR neurons in NS adults was significantly lower than in PHD 35 and PHD 50 juveniles, and the density of intensely-stained FoxP2-IR neurons in PHD 35 juveniles was significantly greater than in PHD 50 juveniles (Tukey's *post-hoc* test, Figure 3E). The density of weakly-stained FoxP2-IR neurons in Area X did not vary significantly across ages, but there was a trend for a lower density observed in PHD 50 juveniles (ANOVA, *p* = 0.0578, *F* = 3.387, Figure 3F), which may be singing related.

### Density of FoxP2-IR neurons in area X decreases exponentially with age in adult male zebra finches

**Figure 4:**
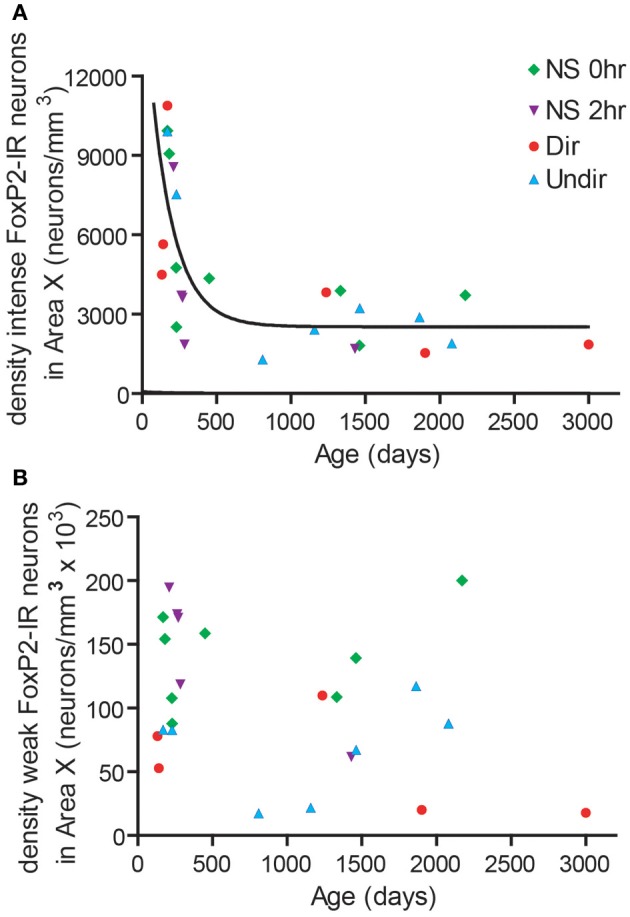
**FoxP2 expression was higher in younger adults than in older adults. (A)** The density of intensely-stained FoxP2-IR neurons followed a one-phase decay relationship relative to age in days (*R*^2^ = 0.5437). **(B)** The density of weakly-stained FoxP2-IR bore no relationship with age (*R*^2^ = 0.1011).

We noticed that the density of intensely-stained FoxP2-IR neurons varied considerably within each group (Figure 2D). The Dir, Undir, and NS groups ranged in ages from PHD 133 to 3000 and included young adults, which sing in a less stereotyped manner than older adults (Pytte et al., 2007) and whose songs degrade much faster after deafening than those of older adults (Lombardino and Nottebohm, 2000). To assess a potential relationship between age and FoxP2-IR density, we compared the age of birds (in days) to the density of FoxP2-IR neurons in Area X in each bird. We found that the density of intensely-stained FoxP2-IR neurons followed an exponential one-phase decay pattern in relation to age (*R*^2^ = 0.5437, Figure 4A). No such relationship was found between density of weakly-stained FoxP2-IR neurons and age (*R*^2^ = 0.1011, Figure 4B).

### Song stereotypy increases with age and is negatively correlated with density of intensely-stained FoxP2-IR neurons in area X

**Figure 5:**
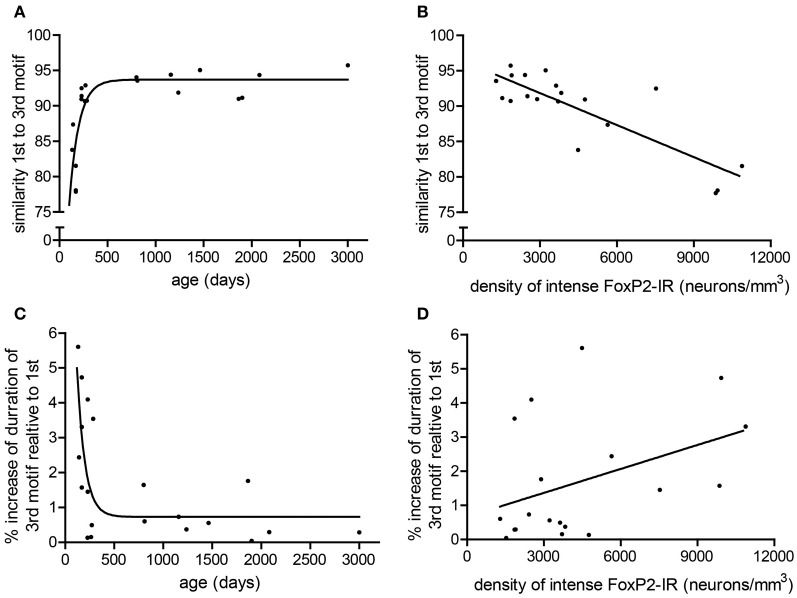
**Measures of stereotypy correlate with age and density of intensely-stained FoxP2-IR neurons. (A)** The similarity of the 1st motif relative to the 3rd motif in a bout followed a one-phase association with age in adult male zebra finches (*R*^2^ = 0.641). **(B)** The similarity of the 1st motif relative to the 3rd motif in a bout was negatively correlated with the density of intensely-stained FoxP2-IR neurons in Area X (*R*^2^ = 0.7131, *p* < 0.0001). **(C)** The ratio of the duration to the 3rd motif relative to the 1st motif in a bout followed a one-phase decay with age in adult male zebra finches (*R*^2^ = 0.4922). **(D)** The ratio of the duration to the 3rd motif relative to the 1st motif in a bout was not significantly correlated with intensely-stained FoxP2-IR neuron density but did illustrate a trend (*R*^2^ = 0.163, *p* = 0.0865).

To determine if consistency of song production across bouts (stereotypy) relates to density of intensely-stained FoxP2-IR neurons, we measured various attributes of song stereotypy. We used the undirected song recordings from the 2 days before the birds were overdosed with anesthesia. Twenty adult males sang sufficient undirected song during this time period for analysis. Regression analyses comparing song measurements to age and intensely-stained FoxP2-IR neuron density are summarized in Table 1. In short, we found that song stereotypy was correlated with either age and/or density of intensely-stained FoxP2-IR neurons. Specifically, the older birds were the more similar they sang the first and third motif in a bout (*R*^2^ = 0.641, Figure 5A) and the lower was the density of intensely-stained FoxP2-IR neurons in Area X (*R*^2^ = 0.7131, *p* < 0.0001, Figure 5B). Consistent with this finding, the duration of the 1st and 3rd motif in strophes was also more similar the older birds were (*R*^2^ = 0.4922, Figure 5C) However, the association of the latter measure of song stereotypy and density of FoxP2-IR neurons missed significance (*R*^2^ = 0.163, *p* = 0.0865, Figure 5D).

**Table 1:** **Statistical results comparing various measures of song stereotypy with age and intensely-stained FoxP2-IR neuron density**.

	**Age**		**FoxP2 Density**	
	*****R***^**2**^**	***p*-value**	*****R***^**2**^**	***p*-value**
**COEFFICIENT OF VARIATION MEASURES**				
Syllable duration	0.0000	0.9772	0.0159	0.6075
Pitch	0.1262	0.1242	0.0098	0.6868
Frequency modulation	0.0399	0.3987	0.1645	0.0849
Amplitude modulation	0.0362	0.4220	0.0143	0.6258
Entropy	0.0307	0.4597	0.0131	0.6407
Pitch goodness	0.0490	0.3484	0.1030	0.1803
Mean frequency	0.0057	0.7516	0.1135	0.1584
Duration 1st motif	0.0773	0.2353	0.0060	0.7529
Duration 3rd motif	0.1037	0.1662	0.0176	0.5878
Intersyllable difference	0.0001	0.9599	0.1449	0.1079
**MOTIF COMPARISONS**				
Similarity 1st to 3rd within bout	0.3032	**0.0119**	0.7131	**>0.0001**
Similarity 1st to 1st across bouts	0.0455	0.3663	0.0169	0.5954
Ratio 1st to 3rd motif duration	0.2685	**0.0192**	0.1630	0.0865
**SONG SEQUENCE MEASURES**				
Linearity	0.0448	0.3706	0.0127	0.6458
Consistency	0.0791	0.2296	0.0908	0.2100
Stereotypy score	0.0574	0.3089	0.0310	0.4708

Some measures of stereotypy varied significantly with density of FoxP2-IR neurons in Area X and with age. We analyzed three categories of stereotypy: coefficient of variation of various song attributes, comparisons across different motifs, and song sequence measures. The R^*2*^ and p-value for each test is listed. Significant differences are indicated in bold text.

### Density of FoxP2-IR cells does not co-vary with amount of singing in adult male zebra finches

**Figure 6:**
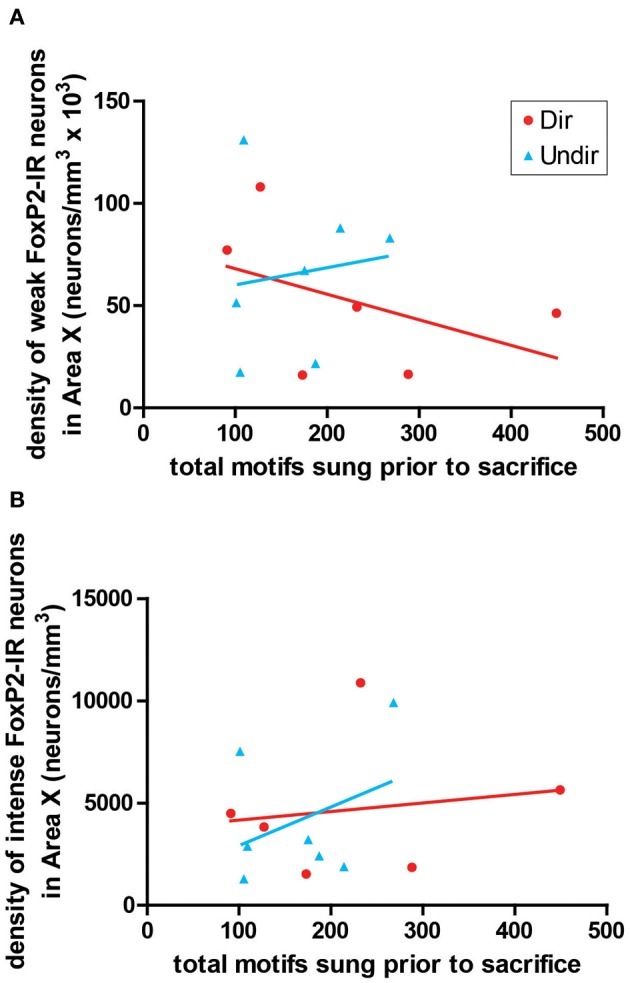
**Expression of FoxP2 did not correlate with level of singing.** The density of weakly-stained **(A)** and intensely-stained **(B)** FoxP2-IR neurons did not co-vary with the number of bouts sung in the 2 h prior to sacrifice in Dir (red circles) and Undir (blue triangles) birds.

To determine if the density of FoxP2-IR correlated with amount of singing, we performed correlation analysis. The density of weakly-stained FoxP2-IR neurons did not significantly co-vary with the number of motifs sung by undirected (*R*^2^ = 0.018, *p* = 0.7722) or directed singers (*R*^2^ = 0.206, *p* = 0.3655, Figure 6A). The same was true for intensely-stained FoxP2-IR (undirected singers *R*^2^ = 0.138, *p* = 0.4123; directed singers *R*^2^ = 0.025, *p* = 0.7627, Figure 6A).

### Density of fusiform FoxP2-IR cells decreases with age in adult male zebra finches

**Figure 7:**
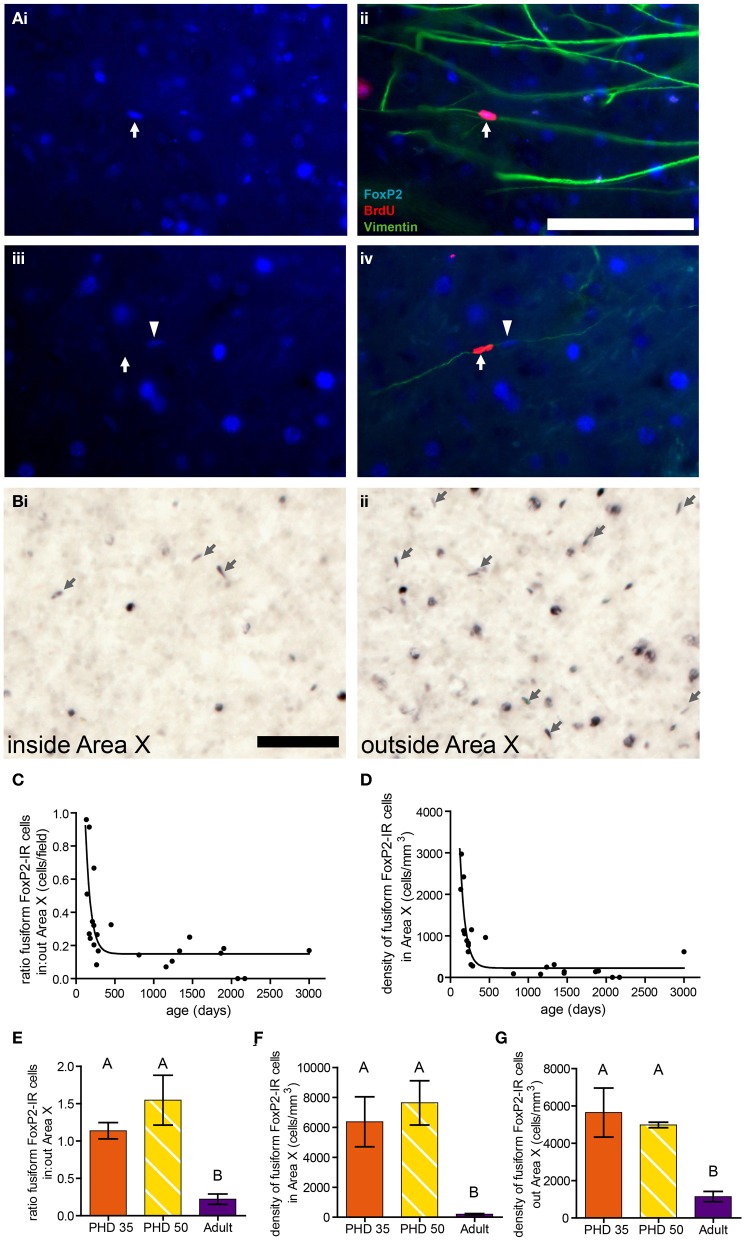
**Fusiform-shaped FoxP2-IR cells associate with vimentin-IR fibers and their density decreases with age.** [**(A)** (i and ii)] Photomicrographs illustrating a fusiform FoxP2-IR (blue) cell, aged 10 days (dated by BrdU injection, red, white arrow) in association with a vimentin-IR (green) radial glia process in the striatum of an adult male. (iii and iv) A fusiform cell which was BrdU-IR but FoxP2-negative (white arrow). Note that next to this cell was a BrdU-negative/FoxP2-IR cell (arrowhead) that was associated with the same vimentin-IR fiber. Scale bar = 100 μm **(B)** Fusiform-shaped FoxP2-IR cells (arrows) inside Area X (i) and outside Area X (ii) in an adult male zebra finch not injected with BrdU. **(C)** The density of fusiform-shaped FoxP2-IR cells in Area X followed a one-phase decay relationship relative to age in days (*R*^2^ = 0.5750). **(D)** The ratio of density of fusiform-shaped FoxP2-IR cells inside Area X relative to outside Area X followed a one-phase decay relationship relative to age in days (*R*^2^ = 0.7926). **(E)** The density of fusiform-shaped FoxP2-IR cells in Area X was significantly higher in juveniles than it is in adults (ANOVA, *p* = 0.0018, *post-hoc* differences indicated by letters above columns). **(F)** The density of fusiform-shaped FoxP2-IR cells outside Area X was significantly higher in juveniles than it is in adults (*p* = 0.0065). **(G)** The ratio of fusiform-shaped cells inside Area X relative to outside Area X was significantly higher in juveniles than in adults (*p* = 0.0105).

In adult birds treated with BrdU to birthdate dividing cells and euthanized 10 days later, we found that ~21% of BrdU-IR cells in the striatum, including Area X, colocalized with FoxP2. These FoxP2-IR/BrdU-IR cells were often fusiform shaped, and the vast majority of these cells were associated with vimentin, which labels radial glial cell processes (Figure 7A), suggesting that the fusiform nuclei belong to migrating new neurons. In a separate group of adults not injected with BrdU, the density of fusiform shaped FoxP2-IR cells was lower inside Area X than in the striatum outside Area X (Figures 7B,C). The ratio of the density of fusiform shaped FoxP2-IR cells inside Area X:outside Area X was higher in younger adult birds and followed a one phase decay (*R*^2^ = 0.5750, Figure 7C). Within Area X, the density of fusiform shaped FoxP2-IR cells decreased as birds got older following a one phase decay (*R*^2^ = 0.7926, Figure 7D). Comparing the ages PHD 35 and PHD 50 to the non-singing adults, we found that the ratio of the density of fusiform shaped FoxP2-IR cells inside Area X:outside Area X was significantly greater in PHD 35 and 50 zebra finches than in adults (ANOVA, *p* = 0.0018, *F* = 15.34, Figure 7E). Likewise, the density of fusiform shaped FoxP2-IR cells inside (Figure 7F) and outside (Figure 7G) Area X was significantly greater in juveniles than in adult zebra finches (ANOVA, *p* = 0.0065, *F* = 10.10, and *p* = 0.0105, *F* = 8.500, respectively).

### Young neurons are more likely to be intensely-stained than weakly-stained for Foxp2

**Figure 8:**
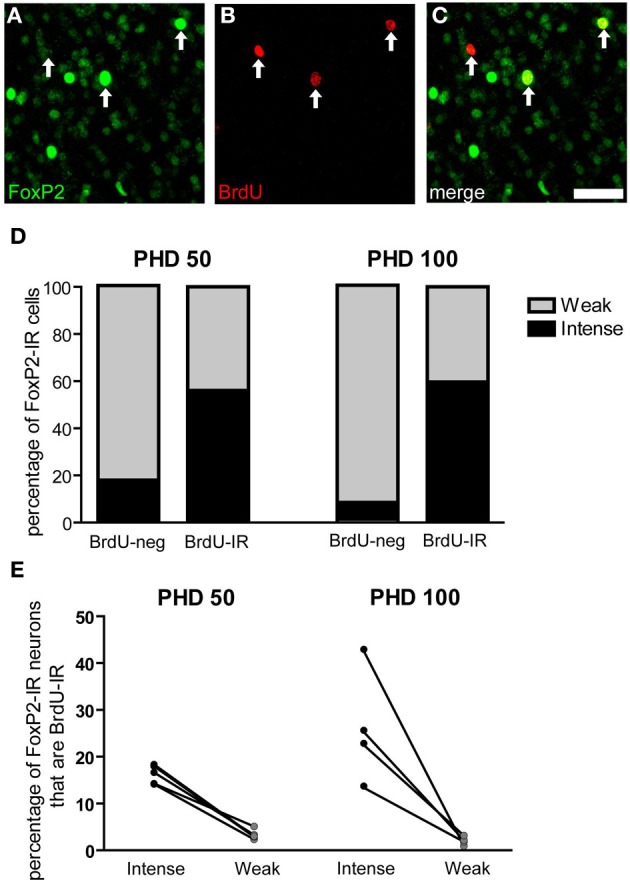
**Three-week old FoxP2-IR neurons are more likely to be intensely-stained than weakly-stained. (A–C)** Photomicrographs depicting fluorescently-labeled FoxP2-IR cells (green) and BrdU-IR cells (red). Arrows indicate cells that are double labeled. Note that the cell on the left is weakly-stained for FoxP2 and the remaining two cells are intensely-stained for FoxP2. Scale bar = 50 μm **(D)** In post-hatch day 50 and 100 males, BrdU-IR cells birth-dated 21 days prior to sacrifice were ~60% intensely-stained for FoxP2, whereas BrdU-negative cells were much less likely to be intensely-stained for FoxP2. **(E)** On average, ~20% of intensely-stained FoxP-IR cells were also BrdU-IR, whereas ~3% of weakly-stained FoxP2-IR cells were also BrdU-IR.

Two groups of male zebra finches were injected with BrdU 21 days before being euthanized at PHD 50 or 100 (Figures 8A–C). More than half of the BrdU-IR/FoxP-IR neurons had intense FoxP2 label in both age groups. In contrast, only 7% of the FoxP2-IR neurons negative for BrdU-IR were intensely FoxP2-immunoreactive at PHD100 (17% at PHD50) (Figure 8D). Fisher's exact test for each individual showed that the percentage of intensely-stained FoxP2-IR neurons that were also BrdU-IR was much higher than would be expected by chance (*p* < 0.01 for all animals analyzed). Moreover, in PHD 50 and 100 finches the percentage of intensely-stained FoxP2-IR neurons in Area X that were also BrdU-IR was 16 and 26%, respectively, whereas the percentage of weakly-stained FoxP2-IR neurons in Area X that were also BrdU-IR was only 3 and 1%, respectively (Figure 8E). Thus, approximately 1/6 to 1/4 of all intensely-stained FoxP2-IR neurons in Area X were born 21 days prior to overdose with anesthesia, and BrdU-IR neurons were more than five times more likely to be intensely-stained for FoxP2 than would be expected by chance.

## Discussion

**Figure 9:**
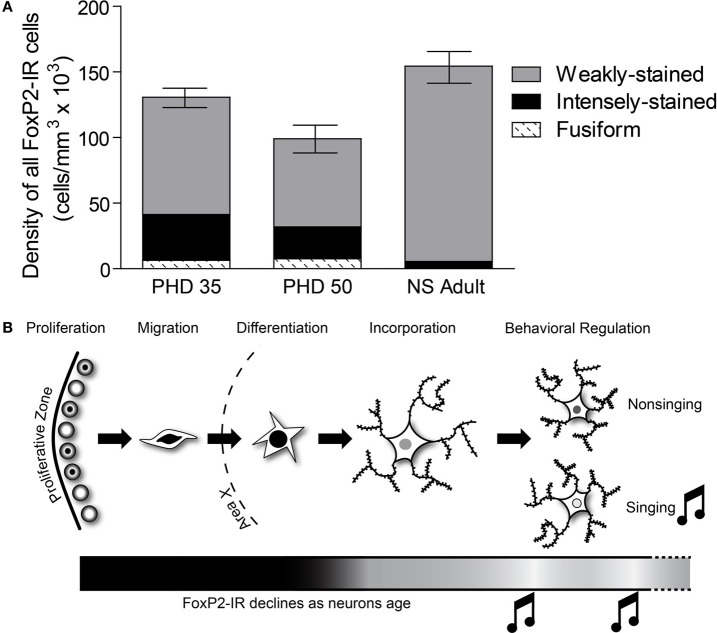
**The relative distribution of the different categories of FoxP2-IR in Area X changes across development. (A)** In PHD 35 and 50 males, nearly one-third of all FoxP-IR cells were either intensely-stained or fusiform-shaped, whereas these categories made up less than 5% of the cells in adults. **(B)** Our data suggests that FoxP2 expression is high in young neurons, in the proliferative zone (Rochefort et al., 2007), during migration, and during differentiation. As neurons are incorporated, FoxP2 expression decreases. Singing behavior temporarily suppresses FoxP2 expression.

Our results support two major conclusions that provide important hints about the function of FoxP2 in shaping cortico-basal ganglia circuits. First, the different intensities of FoxP2-IR observed in zebra finch Area X medium spiny neurons in juveniles and adults suggests the existence of two cell populations within this FoxP2-expressing cell class (Figures 2, 3). We propose that these two classes are subdivisions of the same population, and that the intensity of FoxP2-IR is related to the age of the neuron. We showed that newly incorporated Area X neurons were more likely to be intensely-stained for FoxP2 than would be expected by chance. In fact, between 1/6 and 1/4 of all intensely-stained neurons in Area X were born 21 days prior sacrifice (Figures 8D,E) which suggests that new medium spiny neurons strongly express FoxP2 up to at least 3 weeks after being born in the ventricular zone. The fact that fusiform-shaped, presumably migrating, FoxP2-IR cells became less numerous as animals aged is consistent with the notion that new neurons express more FoxP2 until they are fully incorporated into their neural network (Figure 9). During development of Area X, when new neurons are recruited at higher levels (Rochefort et al., 2007), the density of intensely-stained FoxP-IR neurons was nearly 10 times higher than in adults (Figure 3E). Together, these data suggest that elevated levels of FoxP2 must play an important role during the early events of a medium spiny neuron's life. It was recently shown that during the development of chick and mouse spinal cord, dynamic FoxP2 expression, in concert with FoxP4, is important for delamination from the neuroepithelium and neural progenitor fate maintenance (Rousso et al., 2012). Likewise, knockdown of FoxP2 in mouse embryos inhibits cortical neurogenesis (Tsui et al., 2013). In the zebrafinch, lentiviral-mediated downregulation of FoxP2 in the ventricular zone did not, however, cause a significant reduction of precursor cell division or a noticeable decrease of recruitment into Area X, though it did affect spine density (Schulz et al., 2010). The exact role of FoxP2 during this critical phase of proliferation, migration and incorporation in different species, brain regions, and developmental ages awaits further study.

The second conclusion we draw from our data concerns the behavior-driven regulation of FoxP2 in relation to neuron age. We found that the density of intensely-stained FoxP2-IR neurons decreased in Area X the older the birds were (Figure 4A). This occurred independent of whether or not birds had sung during the 2 h before being euthanized (Figures 2D, 4A). In contrast, the density of weakly-stained FoxP2-IR neurons did not correlate with adult age (Figure 4B) but was lower after singing (Figure 2E). Thus, neurons younger than three weeks are unlikely to contribute to the previously reported singing related reduction of FoxP2 expression in adult Area X (Teramitsu and White, 2006; Miller et al., 2008; Teramitsu et al., 2010).

Our data complement findings from previous reports on FoxP2 expression in Area X tissue that showed higher overall *FoxP2* mRNA expression in Area X at PHD 35 and 50, slightly declining levels by PHD 75, and further decrease after sexual maturation (>PHD 100) (Haesler et al., 2004). This overall pattern of decreasing *FoxP2* mRNA expression also occurs in brains of rodents and macaques (Takahashi et al., 2003, 2008). Teramitsu et al. (2010) reported levels of *FoxP2* mRNA at PHD 50 and PHD 75 to be the same but did not compare these ages to older adult ages. Rochefort et al. (2007) found stable densities of FoxP2-IR neurons throughout development while the number of newborn FoxP2-IR neurons declined. Summing the densities of weakly-stained and intensely-stained FoxP2-IR reveals a similar picture (Figure 9A). Our results show that the major difference between juvenile and adult FoxP2 expression in Area X concerns the *proportion*s of FoxP-IR neurons. Intensely-stained Foxp2-IR, NS regulated neurons become less numerous with age while weakly-stained Foxp2-IR, singing regulated neurons remain detectable at stable densities throughout the bird's life (Figure 9A).

Does this dissociation inform us about the potential role of FoxP2 in Area X function? Area X medium spiny neurons with experimentally reduced FoxP2 levels still build spines but at significantly reduced levels (Schulz et al., 2010). If the deficit of vocal imitation after FoxP2 knockdown in Area X was the result of reduced spine density it would be consistent with FoxP2 playing a molecular role in re-enforcement-based song learning models (Fee and Scharff, 2010; Goldberg and Fee, 2012) that can be extended to mammalian corticostriatal circuits as well (Groszer et al., 2008; Enard et al., 2009; French et al., 2011; Fee, 2012). Reduced numbers of spines in medium spiny neurons may reflect (or lead to) an inability to properly integrate midbrain dopamine signals with sparse song-related signaling from HVC and more variable song-related input via lMAN. Specifically, during initial auditory-motor learning FoxP2 may be needed at high levels in medium spiny neurons to facilitate selecting from the highly variable song those specific song motor patterns that maximize dopamine-mediated reward. Experimental downregulation of FoxP2 at this time would interfere with the matching of particular motor sequences to reward and thus song would not be learned correctly and remain variable, as previously described (Haesler et al., 2007). As song learning progresses, lower FoxP2 levels correlate with the hypothesized consolidation of particular neural microcircuits.

Zebra finch song continues to vary slightly after birds reach sexual maturation, and song is less stereotyped in young adult males for about the first year of life [Brainard and Doupe, 2001; Pytte et al., 2007, but see (Lombardino and Nottebohm, 2000)]. In young adults, similarity of motifs within a bout is decreased and duration of later motifs within a bout is increased (Pytte et al., 2007, Figures 5A,C). Adults continue to monitor their song output and are able to adjust it, if necessary (Brainard, 2004; Tschida and Mooney, 2012); singing seems to be playing an active part in achieving particular song stereotypy levels differing with social context and age. Our observation that the density of weakly-stained FoxP2-IR (older) Area X neurons is highly variable across all ages studied, that it does not correlate with song variability, but is downregulated by adult singing, is consistent with the notion that this class of cells is continuously engaged in maintaining “homeostatic song plasticity.” In other words, FoxP2 in adult animals would continue to exert a role in fine-tuning the selection of inherent (lower) song variability via reenforcement learning. Experimentally lowered FoxP2 levels would thus be expected to interfere with maintenance of adult song as well. Finally, decreasing influx of new neurons as birds age would limit the FoxP2-mediated potential for circuit restructuring and thus result in increasingly stereotyped song. Interestingly, the rate of neuronal recruitment of HVC neurons that synapse onto RA neurons is higher in young adult male zebra finches than in older adults (Wang et al., 1999; Pytte et al., 2007; Walton et al., 2012). These results, in conjunction with our data, suggest that elevated neuronal recruitment into HVC and Area X of juveniles may be an important contributor to song development and song plasticity, and that the reduction of neuronal recruitment may close the critical period and facilitate greater song stereotypy. Future experiments would have to selectively modify neuronal recruitment in HVC and Area X in order to disambiguate the relative contributions of each to song stability. While these ideas seem plausible, they are certainly only a few of various scenarios how our current knowledge about the different levels of FoxP2 expression in relationship to song behavior can be consolidated conceptually.

Both directed and undirected song reduced FoxP2-IR in the present study (Figures 2D,E) as was previously shown for FoxP2 protein levels in Area X by Western blot (Miller et al., 2008). This contrasts with *FoxP2* mRNA expression, which is only affected by undirected singing (Teramitsu and White, 2006). It is possible that social context affects transcription, translation, and protein degradation differentially, or that the amount of singing plays a role. In the mRNA study (Teramitsu and White, 2006) the undirected singers sang many more bouts than did the directed singers. In the protein studies (Miller et al., 2008 and Figure 6) the directed and undirected groups sang similar amounts. Regardless of the explanation, a study controlling the amount of singing over a very narrow time period and sacrificing animals at several time points subsequent to the singing period could resolve this issue.

Does singing also affect the density of weakly-stained Foxp2-IR neurons during development of Area X? Singing behavior decreases the overall expression of *FoxP2* mRNA in Area X in PHD75 male zebra finches (Teramitsu et al., 2010). We did not record singing behavior of juveniles prior to sacrifice but the substantially (though not statistically significant) lower density of FoxP-IR at PHD 50 than at PHD 35 or in NS adults (Figure 3F) may have been due to a single strong singer in the PHD 50 group. PHD 50 males sing more frequently than PHD 35 males (Slater and Jones, 1998; Johnson et al., 2002), so the PHD 35 males are likely to have sung very little, if at all, prior to sacrifice, which could explain why the density of weakly-stained FoxP-IR neurons is relatively high in this group. Future experiments should address the influence of song rate in juveniles, as well as hearing (Teramitsu et al., 2010), on weakly-stained FoxP2 neurons, because it will help to either falsify or support the above hypothesis about FoxP2 function in activity-driven selection of song motor gestures. According to our hypothesis, singing-driven downregulation of FoxP2 in Area X would not be expected to occur before the HVC axons start synapsing onto medium spiny neurons and ascending dopamine innervation converges at the same time. Addressing the relationship between FoxP2 and the suite of other singing-regulated genes (Jarvis and Nottebohm, 1997; Jarvis et al., 1998; Velho et al., 2005; Hilliard et al., 2012) in Area X will help clarify the role FoxP2 plays in medium spiny neuron function.

Our results may shed some light on the role of FoxP2 in shaping neural circuits in mammals as well. The overwhelming consensus is that postnatal neurogenesis in mammalian central nervous system is mostly, if not completely, limited to the hippocampus and the olfactory bulb, a conclusion that largely based on results from adult mice and rats. Postnatal neurogenesis is not nearly as compartmentalized as is widely believed, however. In juvenile mice, GABAergic neurons arising from the neurogenic subventricular zone are incorporated into many parts of the brain, including the cortex and the striatum (Inta et al., 2008). In adult rabbits, new neurons are incorporated into the caudate nucleus, though it was not conclusively shown that these new neurons are medium spiny neurons (Luzzati et al., 2006). Even though the extent of striatal neurogenesis in adolescent humans is not known, neurogenesis in the subventricular zone is substantially reduced by 18 months of age (Sanai et al., 2011); this result suggests that changes in neuronal recruitment in the striatum are unlikely to play a major role in language development. Given FOXP2's role in language development in humans, it may be the case that medium spiny neurons have very high levels of FOXP2 expression in adolescent humans, and that FOXP2 expression may decline as humans age. It is interesting to note that humans that are isolated from a normal language environment for the first ~10 years of life develop only rudimentary language capabilities (Singh et al., 1966; Curtiss, 1977), similar to how isolated songbirds fail to develop normal song as adults (Konishi, 1965). Perhaps the closing of the “critical period” in language acquisition in humans is mediated by a similar age-related decline in the number of striatal medium spiny neurons intensely expressing FOXP2. In fact, it may be the case that, like songbirds, age-related changes in FOXP2 expression are specific to striatal areas that are important for language (Teichmann et al., 2008) and that non-language areas may not have pronounced age-related changes in expression of FOXP2.

In conclusion, our study raises the possibility that a decrease in the recruitment of intensely-stained FoxP2-IR neurons as vocal learners age may affect their vocal flexibility and may therefore be a key event in the ability to properly imitate a vocal template.

### Conflict of interest statement

The authors declare that the research was conducted in the absence of any commercial or financial relationships that could be construed as a potential conflict of interest.

## Footnotes

^1^Following nomenclature proposed by Kaestner and colleagues (1). Upper case (FOXP2) and lower case (FoxP2) refer to human and non-human transcription factors, respectively. Italicized letters (FOXP2 and FoxP2) refer to the respective genes.
